# Lost and found! Tooth fragment reattachment after 8 Mo of trauma

**DOI:** 10.1002/ccr3.2463

**Published:** 2019-10-03

**Authors:** Sudheesh Mani Kakkunath, Nandini Kumari Katta, Ravi Shankar Yelamanchi, Deepthi Mandava

**Affiliations:** ^1^ Department of Oral Surgery Faculty of Dentistry AIMST University Bedong Malaysia; ^2^ Department of Paediatric Dentistry Faculty of Dentistry AIMST University Bedong Malaysia; ^3^ Department of Dental Biomaterials Faculty of Dentistry AIMST University Bedong Malaysia; ^4^ Department of Conservative Dentistry and Endodontics Faculty of Dentistry AIMST University Bedong Malaysia

**Keywords:** fracture, reattachment, tooth fragment, trauma

## Abstract

Thorough clinical and radiographic evaluation of patient following orofacial trauma is indispensable for a successful outcome, and when the tooth fragment is available in a good condition, then fragment reattachment is the best choice of treatment.

## Graphical abstract

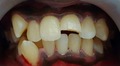

## 1 CASE PRESENTATION

**Figure 1:**
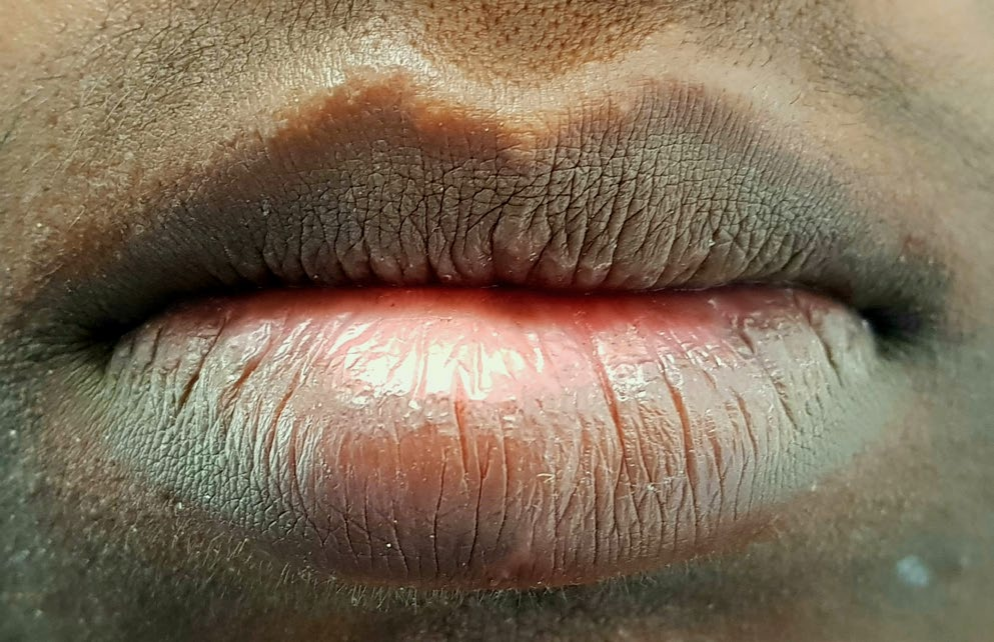
Extraoral lip swelling

**Figure 2:**
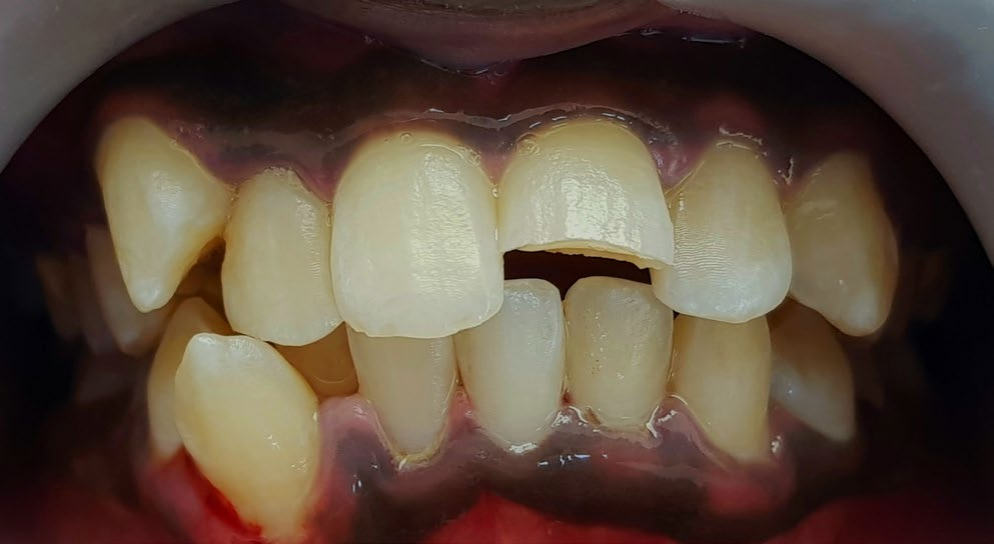
Ellis class II fracture with tooth 21

**Figure 3:**
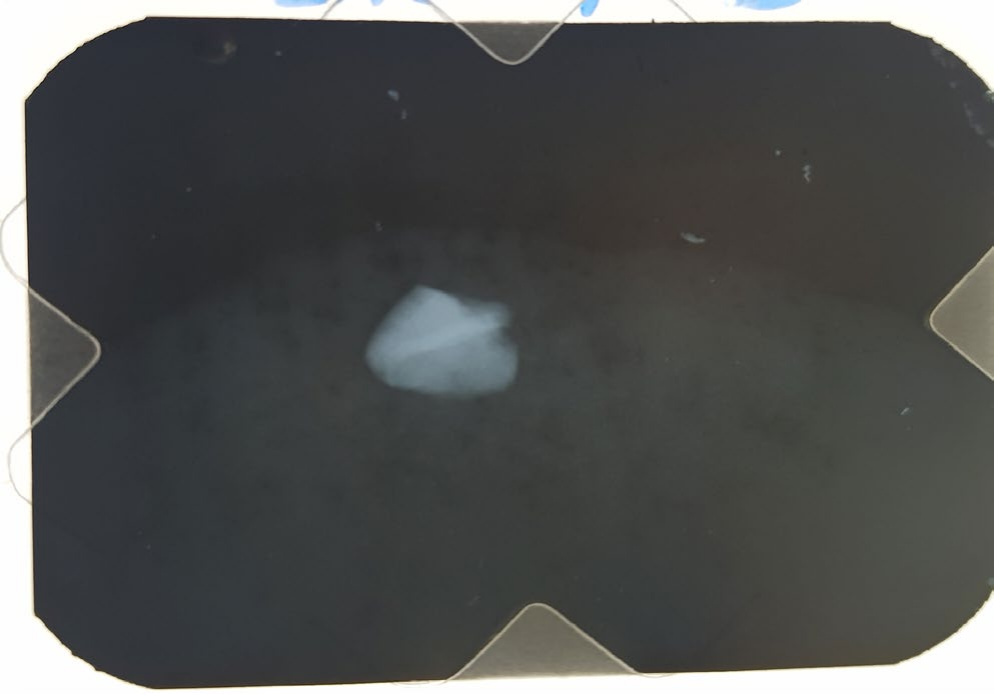
Extraoral radiograph of lower lip

**Figure 4:**
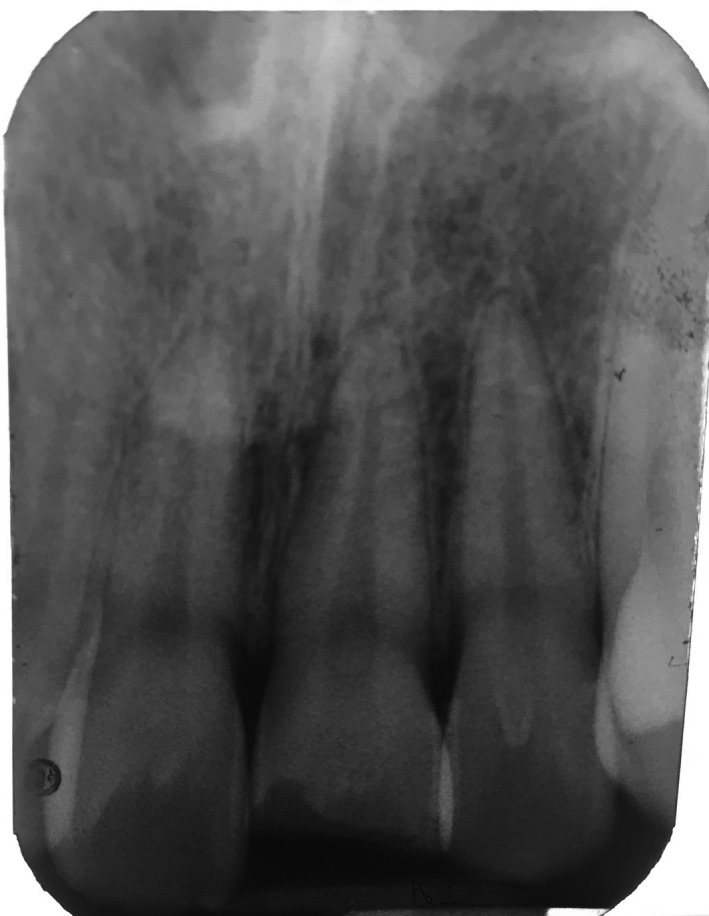
IOPA of tooth 21

**Figure 5:**
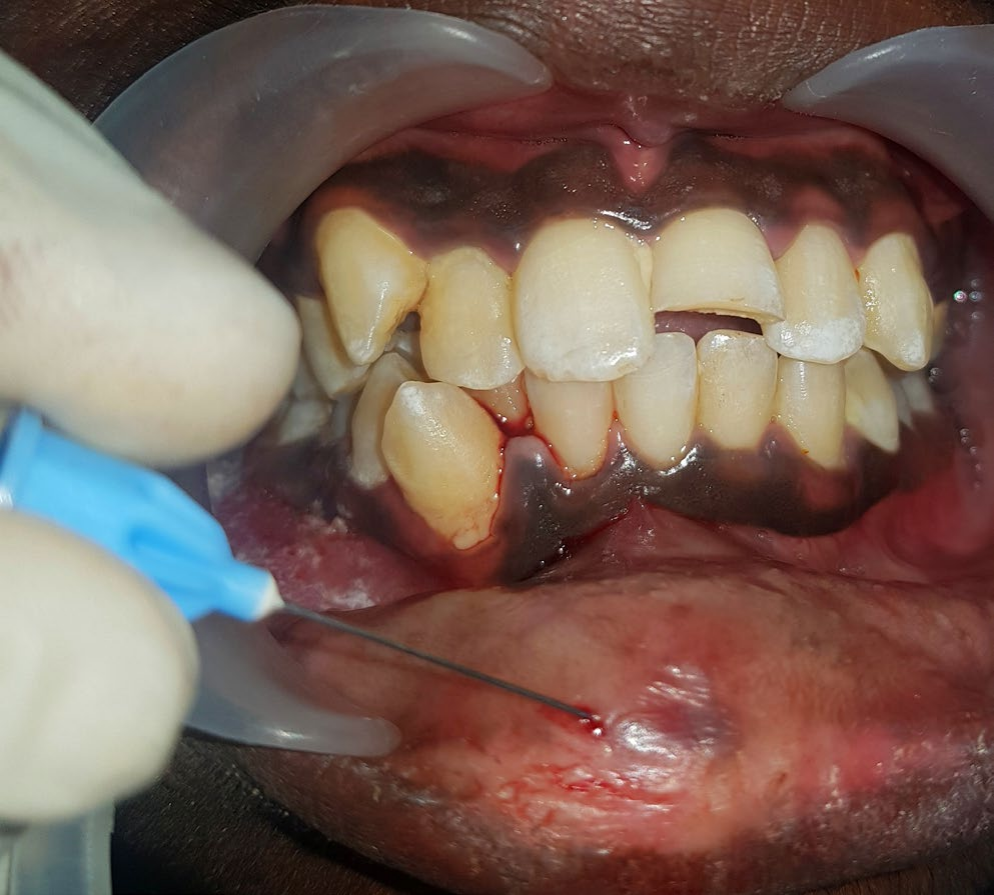
Fragment location using a 25 gauge needle

**Figure 6:**
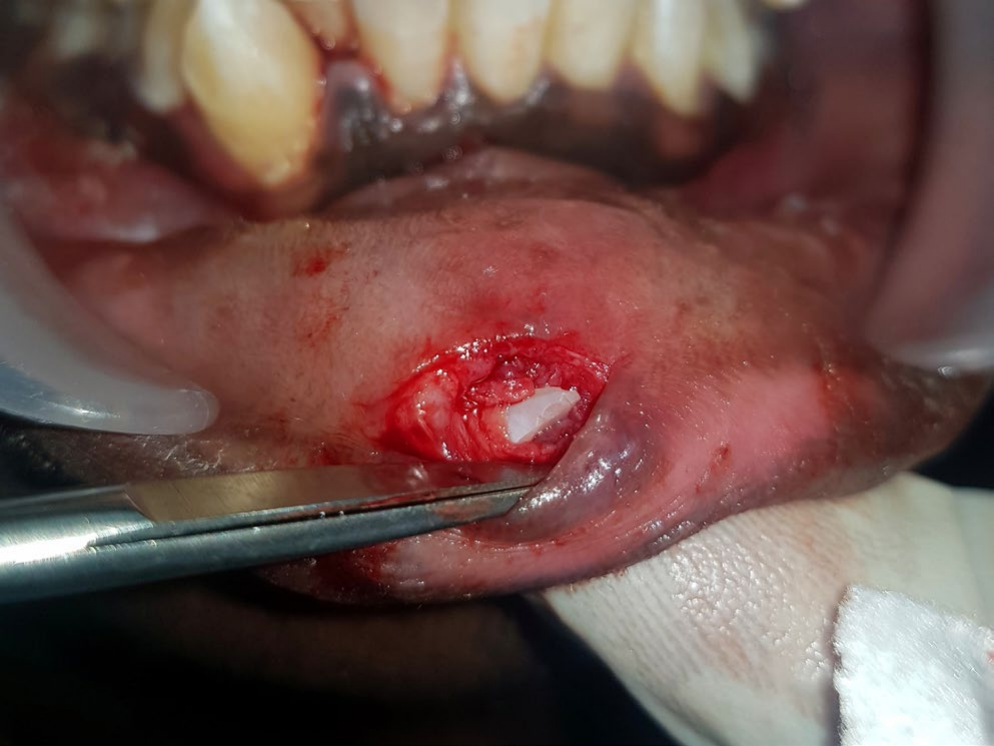
Blunt dissection showing tooth fragment

**Figure 7:**
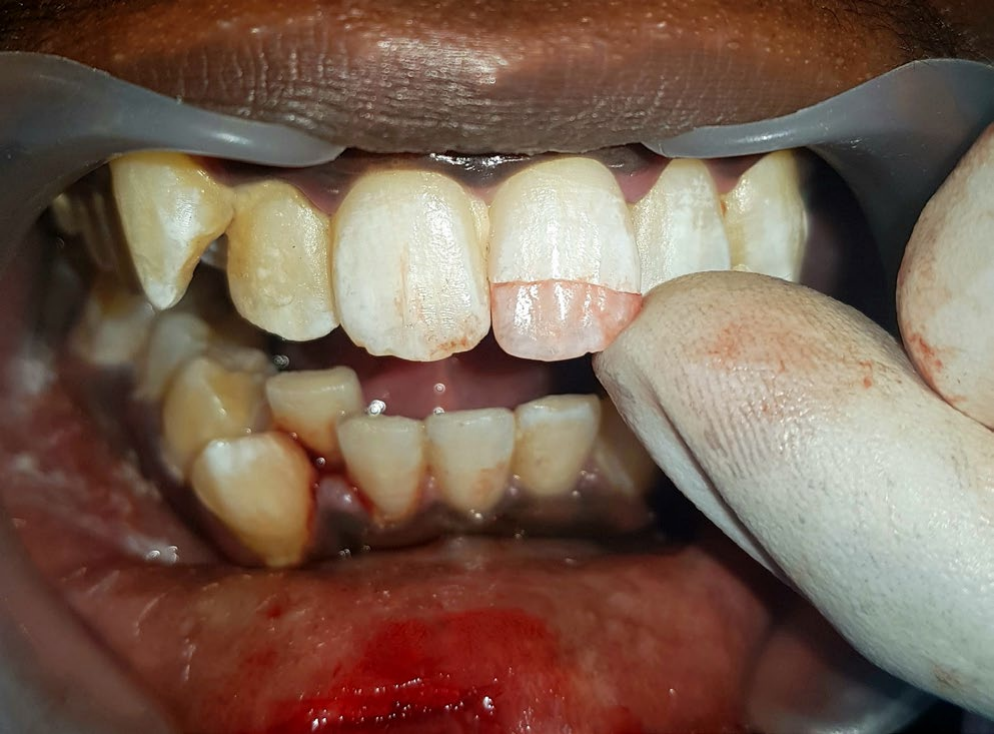
Approximation of tooth fragment

**Figure 8:**
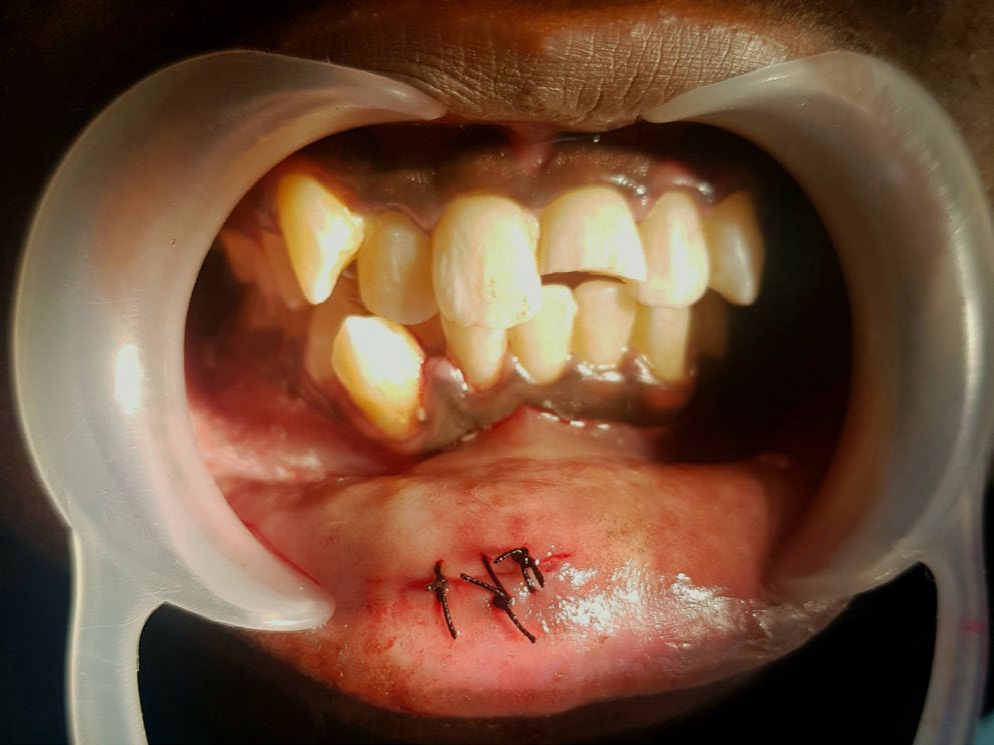
Wound closure done

**Figure 9:**
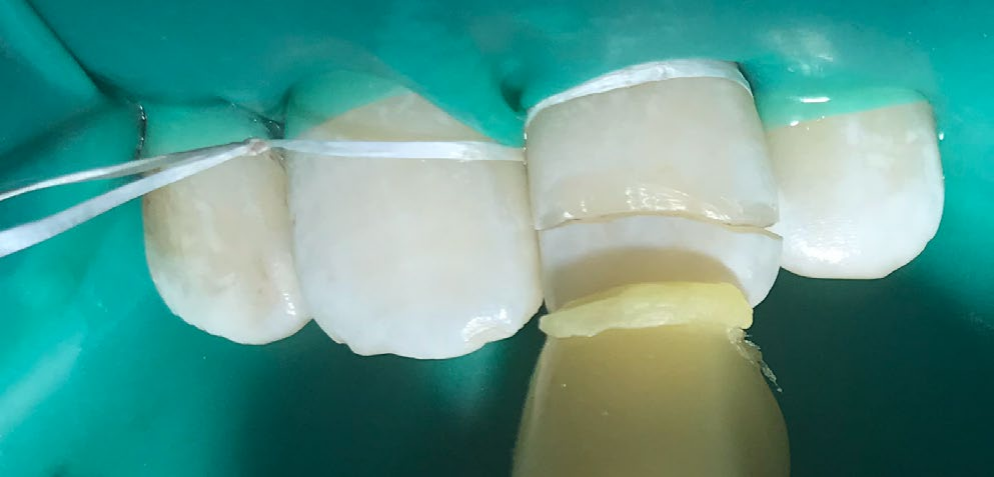
Approximation of tooth fragment with sticky wax

**Figure 10:**
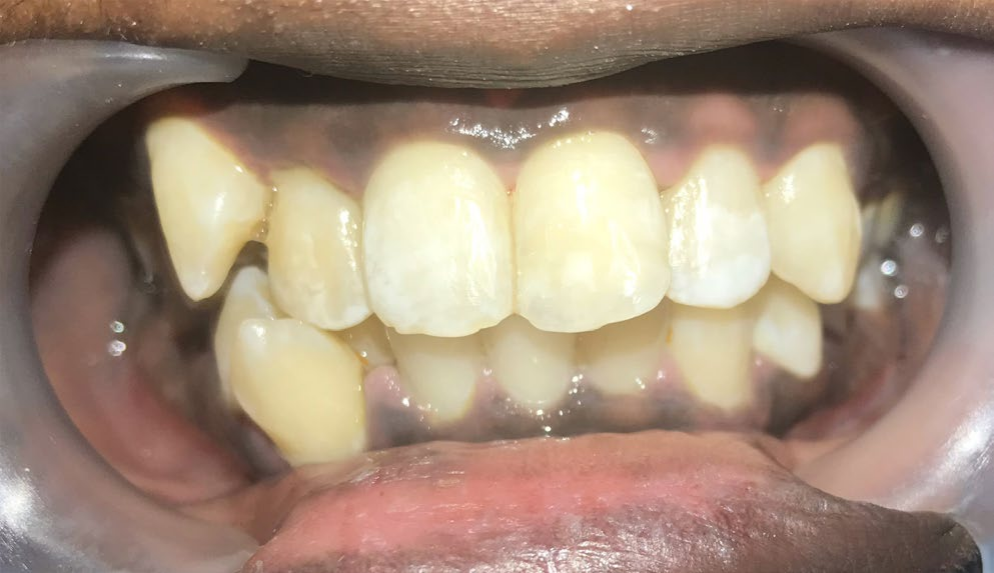
Reattachment of tooth fragment

**Figure 11:**
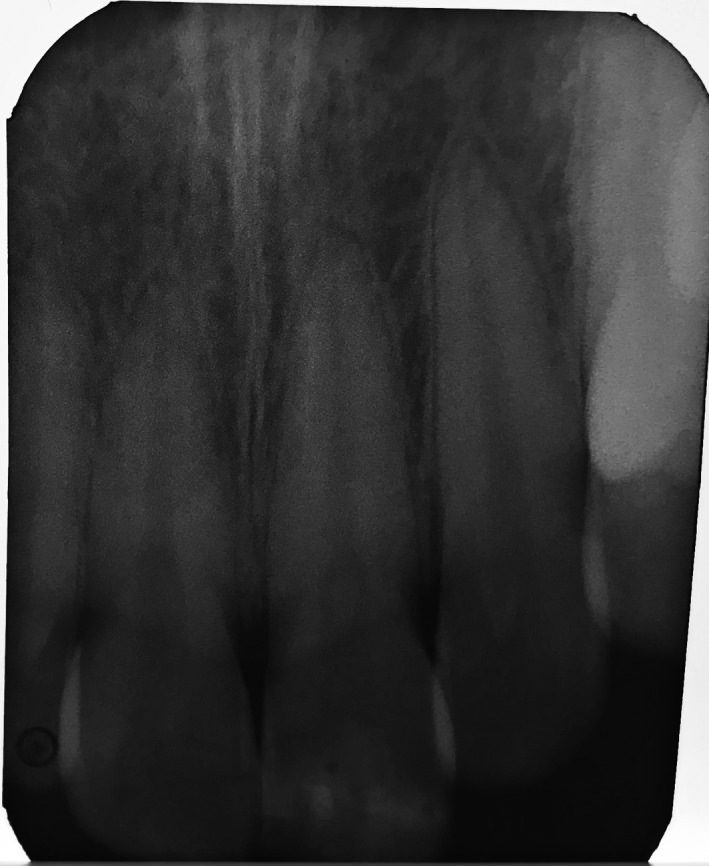
Post‐op IOPAR

A 16‐yr‐old patient reported with a complaint of swollen lower lip for past 1 month. Patient's medical history revealed trauma to front tooth 8 months ago. Figure 1 shows diffuse swelling on the right side of the lower lip with a scar. A nontender mass was palpable underneath the scar. Figure 2 reveals an Ellis class II fracture with tooth 21. Occlusal film (Figure 3) showed the presence of a suspected radiopaque mass resembling a tooth fragment. IOPA of tooth 21 (Figure 4) showed no evidence of damage to the pulp and the periodontium. After proper consent, the fragment was located using a 25 gauge 20‐mm short needle (Figures 5 and 6). The retrieved fragment was checked for approximation with the fractured tooth 21 (Figure 7) and was subsequently stored in normal saline (Figure 8). After suture removal, slight beveling of the fractured edge was done and an adhesive system (Adper Single Bond Plus, 3M ESPE) was used to reattach the tooth fragment with composite resin (Z‐100, 3M ESPE).^1^, ^2^ (Figures 9, 10, 11)^ ^.

Thorough clinical and radiographic evaluation of patient following orofacial trauma is indispensable for a successful outcome, and when the tooth fragment is available in a good condition, then fragment reattachment is the best choice of treatment.

## CONFLICT OF INTEREST

There are no conflicts of interest.

## AUTHOR CONTRIBUTIONS

First author: examined the patient, addressed the chief complaint, located the tooth fragment, and helped in surgical retrieval of the same. Second Author: reattached the tooth fragment. Third Author: edited and proofread the manuscript. Fourth Author: reattached the tooth fragment.
